# Crystal structure of poly[tetra-μ-chlorido-tetra­chlorido­bis­(μ_3_-4,4′-bi-1,2,4-triazole-κ^3^ *N* ^1^:*N* ^2^:*N* ^1′^)(μ-4,4′-bi-1,2,4-triazole-κ^3^ *N* ^1^:*N* ^1′^)tetra­copper(II)]

**DOI:** 10.1107/S2056989019005516

**Published:** 2019-05-14

**Authors:** Kostiantyn V. Domasevitch, Andrey B. Lysenko

**Affiliations:** a Inorganic Chemistry Department, Taras Shevchenko National University of Kyiv, Volodimirska Street 64, Kyiv 01033, Ukraine

**Keywords:** crystal structure, 4,4′-bi-1,2,4-triazole, metal–organic frameworks, copper(II) complexes

## Abstract

The title Cu^2+^–chloride coordination polymer with the 4,4′-bi-1,2,4-triazole ligand (btr), [Cu_4_Cl_8_(C_4_H_6_N_6_)_3_]_*n*_, crystallizes in the non-centrosymmetric ortho­rhom­bic space group F*dd*2. The two independent Cu^2+^ cations adopt distorted square-pyramidal geometries with {Cl_2_N_2_+Cl} coordination polyhedra. The metal atoms are bridged by *μ*-Cl anions forming left- and right-handed helical chains of sequence [–(*μ*-Cl)CuCl–]_*n*_ along the *c-*axis direction. In the perpendicular directions, the btr ligands act in *μ*- and *μ*_3_– coordination modes in a 2:3 ratio. The *μ*-btr bridges connect neighboring helices of the same handedness, whereas the *μ*_3_-btr ligands link the helices of opposite handedness, leading to a racemic three-dimensional framework. The structure is consolidated by weak C—H⋯Cl and C—H⋯N inter­actions.

## Abstract 2

The self-assembly of Cu^2+^ cations, chloride anions and 4,4′-bi-1,2,4-triazole) (C_4_H_4_N_6_; btr) in aqueous solution resulted in a new three-dimensional coordination polymer, which was structurally characterized. A characteristic feature of the coordination framework is the presence of [–(*μ*-Cl)CuCl–]_*n*_ helical chains inter­linked by the btr ligands.

## Chemical context   

4,4′-Bi-1,2,4-triazole, C_4_H_4_N_6_, btr, represents a unique example of a bitopic ligand used for the design of coordination solids. Four nitro­gen donor sites in the btr mol­ecule provide the possibility of different bridging modes [*e.g*. bi-N1,N1′ (Liu *et al.*, 2007 ▸), bi-N1,N2 (Zhang *et al.*, 2008 ▸) tri-N1,N2,N1′ (Huang, Zhao *et al.*, 2008 ▸) and tetra­dentate N1,N2,N1′,N2′ (Lysenko *et al.*, 2006 ▸)], generating extended coordination networks. In this context, small nucleophilic anions play a very important role in the formation of the [*M*–*X*–*M*]_*n*_ coordination units (*X* = OH,^−^ Cl^−^ and Br^−^) that often function as secondary building blocks. In this case, the tri- and tetra­dentate behavior of btr can be preferably realized (Lysenko *et al.*, 2006 ▸, 2007 ▸). Indeed, the CuCl_2_–btr system is very sensitive to the reaction conditions. For example, a one-dimensional coordination polymer of [Cu_3_(*μ_2_*-Cl)_2_Cl_2_(btr)_4_]Cl_2_ was isolated from an aqueous solution (Lysenko *et al.*, 2006 ▸). Another one-dimensional coordination polymer of [Cu(*μ_2_*-Cl)_2_(btr)]·H_2_O was isolated in the presence of aqueous HCl (Zhang *et al.*, 2008 ▸). In this paper, we report the crystal structure of the title three-dimensional coordination polymer, (I), which was also prepared from aqueous solution by mixing CuCl_2_, btr and NH_4_Cl.

## Structural commentary   

The title compound crystallizes from aqueous solution in the ortho­rhom­bic system, non-centrosymmetric space group F*dd*2. The asymmetric unit consists of two copper(II) atoms, four chloride anions and one and a half crystallographically independent btr mol­ecules. One btr ligand occupies a general position, while a half of btr sits on a special position (2-twofold axis running along the *c* axis, perpendicular to the N—N single bond).
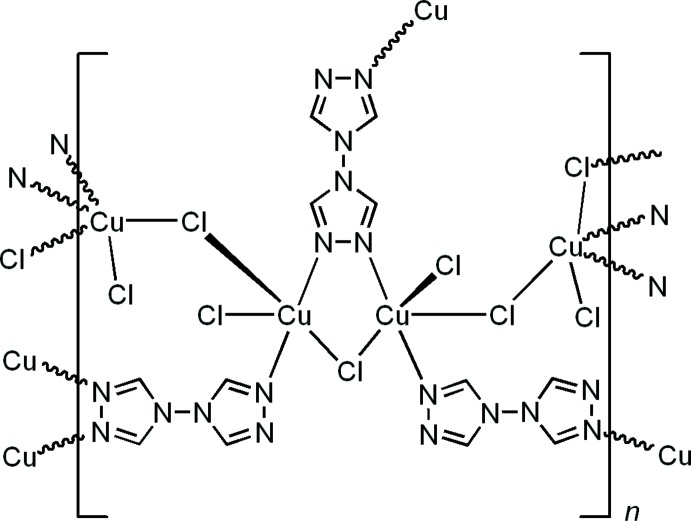


**Figure 1:**
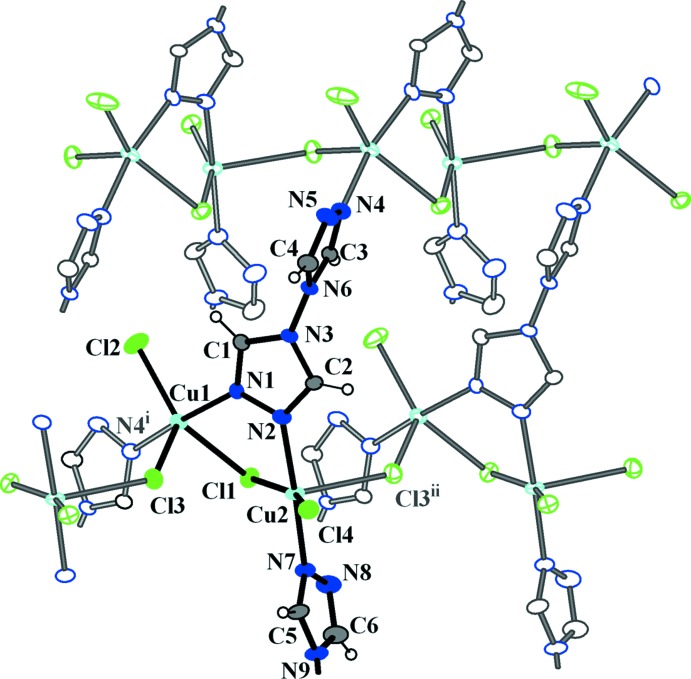
A portion of the structure of (I), showing the atom-labeling scheme and the copper coordination environments. Displacement ellipsoids are drawn at the 50% probability level. [Symmetry codes: (i) *x* − 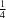, −*y* + 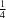, *z* + 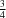; (ii) *x*, *y*, *z* − 1].

**Figure 2:**
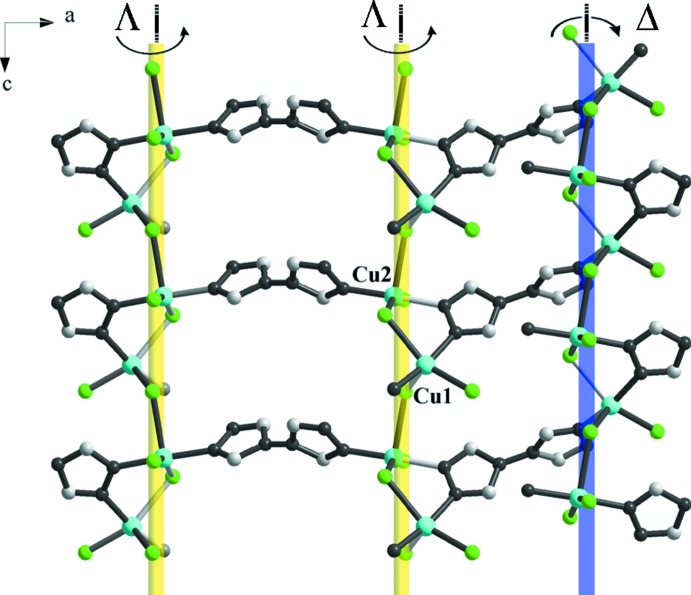
A portion of the helical structure of (I) (view in the *ac* plane). The *μ*-btr mol­ecules link two neighboring helices of the same handedness, whereas the *μ*_3_-btr mol­ecules link two neighboring helices of the opposite handedness. Hydrogen atoms are omitted for clarity.

**Figure 3:**
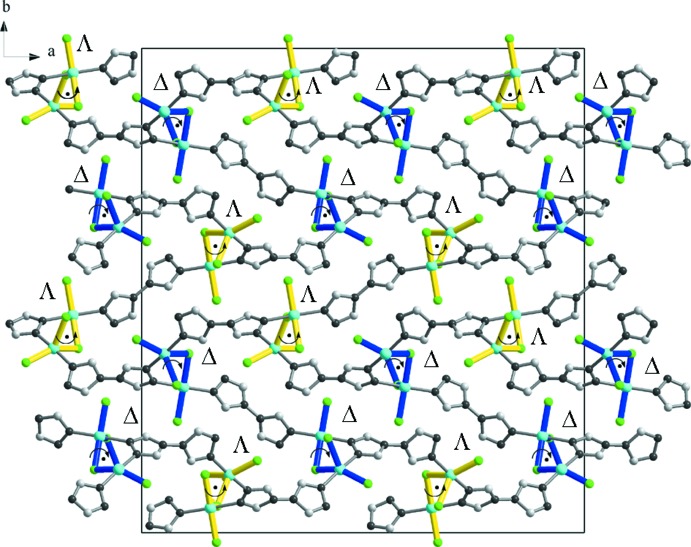
The three-dimensional helical framework structure of (I) (top view).

The first copper ion, Cu1, adopts a distorted square-pyramidal {Cl_2_N_2_+Cl} coordination with two triazole N atoms and two chloride anions in the plane [Cu1—N1 = 1.985 (3) Å, Cu1—N4^i^ = 1.957 (3) Å, N4^i^—Cu1—N1 = 168.82 (15)° symmetry code: (i) *x* − 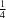, −*y* + 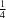, *z* + 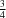, and Cu1—Cl2 = 2.2780 (12) Å, Cu1—Cl1 = 2.5146 (11) Å] and one chloride co-ligand at the apical position [Cu1—Cl3 = 2.4155 (10) Å, Fig. 1 ▸, Table 1 ▸]. Addison *et al.* (1984 ▸) introduced the geometric parameter τ to distinguish whether the geometry of five-coordinate systems is square-pyramidal or trigonal–bipyramidal. According to this scheme, trigonal–bipyramidal geometries are associated with a τ value close to 1.00, whereas for square-pyramidal geometries this value is around 0. Here, the value of τ for Cu1 is 0.35, suggesting the coordination is closer to square-pyramidal. The second independent copper cation, Cu2, has a similar square-pyramidal coordination geometry {Cl_2_N_2_+Cl} with τ = 0.32. Two triazole nitro­gen atoms (N2, N7) and two chloride anions (Cl1, Cl4) comprise the basal plane whereas the fifth chloride donor [Cl3^ii^, symmetry code: (ii) *x*, *y*, *z* − 1] occupies an apical site. The copper polyhedra are linked together through the μ_*2*_-bridging Cl1 and Cl3 anions to form left- and right-handed [Cu1–Cl1–Cu2–Cl3]_*n*_ helices running along the *c-*axis direction (Fig. 2 ▸). The helices have a straight line helical axis (2_1_ axis), with the pitch being equal to the lattice parameter *c*. The btr ligands adopt *μ*- and *μ*_3_- coordination modes in a 2:3 ratio. It is inter­esting to note that the *μ*-bridge btr mol­ecules connect two neighboring helices of the same handedness (ΔΔ or ΛΛ). Then, each helix is connected to the other two of opposite handedness through *μ*_3_-bridging btr mol­ecules, thus forming a three-dimensional framework structure (Fig. 3 ▸). The btr ligand conformation is characterized by a torsion angle between its triazole planes. The *μ*- and *μ*_3_-btr ligands are twisted around the N—N single bond adopting a non-coplanar orientation of the triazolyl groups. The dihedral angles between two triazolyl rings are 74.4 (2) and 78.1 (2)° for *μ*-and *μ*_3_-btr, respectively.

## Supra­molecular features   

**Figure 4:**
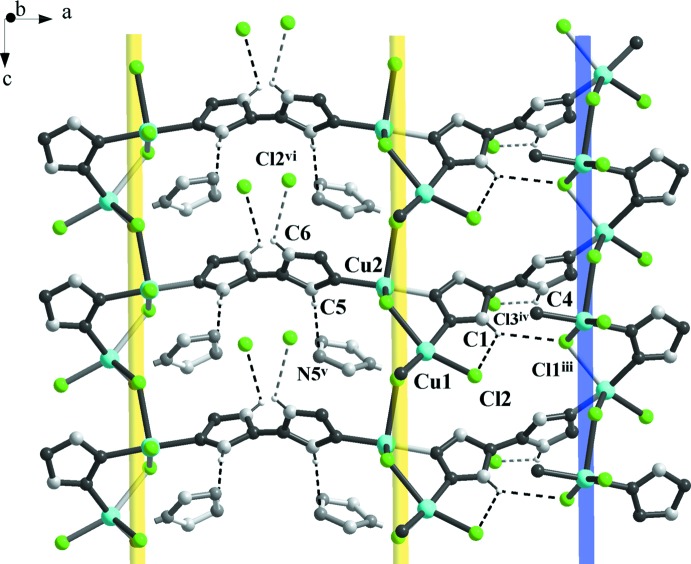
The packing of (I) (view along the [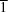
51] direction), showing the non-classical C—H⋯Cl and C—H⋯N hydrogen-bonded inter­actions that support the three-dimensional coordination framework. Hydrogen bonds are shown as dashed lines. [Symmetry codes: (iii) 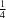 + *x*, 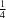 − *y*, 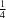 + *z*, (iv) 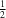 − *x*, −*y*, −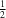 + *z*, (v), 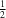 − *x*, −*y*, 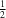 + *z*, (vi) −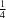 + *x*, 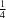 − *y*, −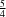 + *z*].

In the crystal, compound (I) exhibits non-classical C—H⋯Cl and C—H⋯N hydrogen bonds (Fig. 4 ▸, Table 2 ▸). The C5 carbon atom of the triazole ring, as a weak hydrogen-bond donor (Desiraju & Steiner, 1999 ▸), is involved in a hydrogen bond with the acceptor N5^v^ atom of the neighboring triazole fragment. There is a bifurcated contact between one C1—H1 fragment and Cl2 (major component) and Cl1^iii^ (minor component). Two other hydrogen-bonding inter­actions are found between the C4—H4 and C6—H6 fragments and atoms Cl3^iv^ and Cl2^vi^, respectively.

In conclusion, the study demonstrates that a combination of a neutral btr mol­ecule and a chloride anion, as complementary donor units, has promising potential in the development and design of metal–organic frameworks.

## Database survey   

According to our CSD search (version 5.39, update May 2018; Groom *et al.*, 2016 ▸), the ligand geometries in (I) are in agreement with a general tendency for the coordinating btr ligand to adopt a twisted conformation. The only exception was observed for the Mn^II^–oxalate complex [Mn_2_(btr)(C_2_O_4_)_2_(H_2_O)_2_]·2H_2_O (Huang & Cheng, 2008 ▸), in which the torsion angle is close to 0°. In the pure ligand, the dihedral angle is equal to *ca* 88° (Domiano, 1977 ▸).

## Synthesis and crystallization   

4,4′-Bi-1,2,4-triazole (btr) was prepared in a yield of 60% by the literature transamination reaction between 4-amino-1,2,4-triazole and *N*,*N*-di­methyl­formamide azine (Bartlett & Humphrey, 1967 ▸).

A solution of CuCl_2_·2H_2_O (34.0 mg, 0.20 mmol) and NH_4_Cl (10.6 mg, 0.20 mmol) in 2 ml of water was added to a solution of btr (27.2 mg, 0.20 mmol) in water (0.5 ml). A drop of 0.10 *M* HCl aqueous solution was then added. The resulting green solution was left standing for several days to form green prismatic crystals. The product was filtered, washed with water and dried in air (yield 47%). Analysis calculated for C_12_H_12_Cl_8_Cu_4_N_18_ (I): C, 15.23; H, 1.28; N, 26.65%. Found: C, 15.20; H, 1.32; N, 26.55. IR (KBr disks, selected bands, cm^−1^): 608*s*, 668*w*, 856*m*, 896*w*, 950*w*, 1022*s*, 1044*s*, 1076*m*, 1102*m*, 1212*w*, 1308*m*, 1338*w*, 1354*w*, 1400*w*, 1498*m*, 1536*m*, 3088*s*, 3112*s*, 3120*s*.

**Figure 5:**
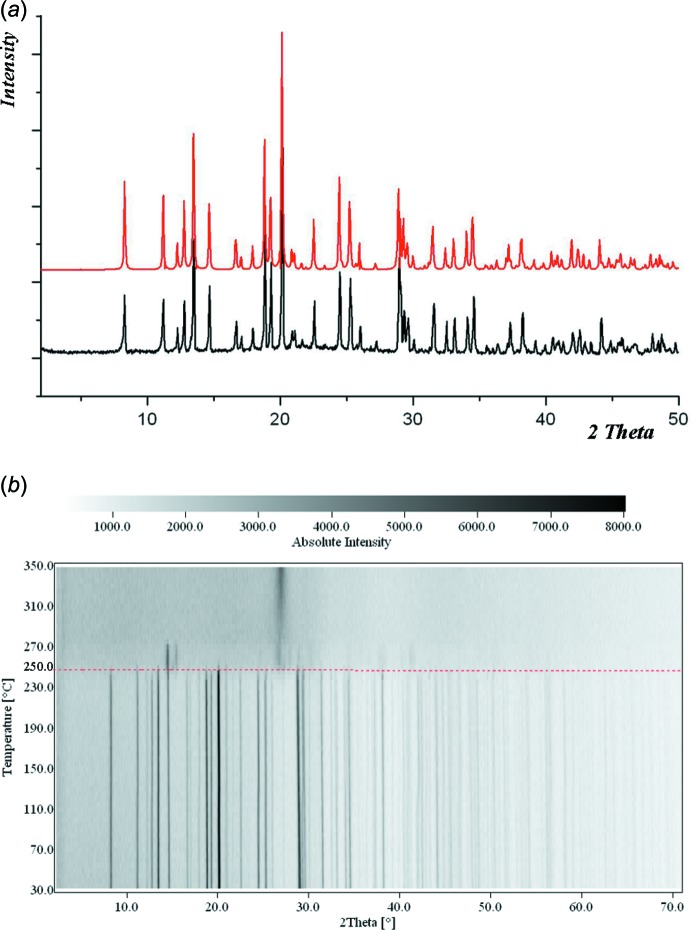
(*a*) PXRD data [calculated (red line) and experimental (dark line)] and (*b*) two-dimensional thermo-PXRD patterns for (I) (Cu *K*_α1_ radiation).

The thermal stability of (I) was investigated by measurements of temperature-dependent PXRD (Fig. 5 ▸). In the temperature-dependent X-ray diffractograms, the initial positions of the main diffraction peaks remain unchanged upon heating to 523 K. Above this temperature, the compound undergoes irreversible thermal decomposition, resulting in an amorphous solid.

## Refinement   

Crystal data, data collection and structure refinement details are summarized in Table 3 ▸. All C-bound H atoms were placed at calculated positions [C—H = 0.94 Å (aromatic)] and refined using a riding model with *U*_iso_(H) = 1.2*U*_eq_(CH).

## Supplementary Material

Crystal structure: contains datablock(s) I. DOI: 10.1107/S2056989019005516/hb7819sup1.cif

Structure factors: contains datablock(s) I. DOI: 10.1107/S2056989019005516/hb7819Isup2.hkl

CCDC reference: 1911618

Additional supporting information:  crystallographic information; 3D view; checkCIF report

## Figures and Tables

**Table 1:** Selected bond lengths (Å)

Cu1—N4^i^	1.957 (3)	Cu2—N7	2.031 (3)
Cu1—N1	1.985 (3)	Cu2—N2	2.032 (3)
Cu1—Cl2	2.2780 (12)	Cu2—Cl4	2.2769 (9)
Cu1—Cl3	2.4155 (10)	Cu2—Cl1	2.3185 (10)
Cu1—Cl1	2.5146 (11)	Cu2—Cl3^ii^	2.6238 (13)

Symmetry codes: (i) 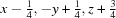; (ii) 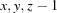.

**Table 2:** Hydrogen-bond geometry (Å, °)

*D*—H⋯*A*	*D*—H	H⋯*A*	*D*⋯*A*	*D*—H⋯*A*
C1—H1⋯Cl1^iii^	0.94	2.74	3.528 (4)	142
C1—H1⋯Cl2	0.94	2.53	3.052 (4)	115
C4—H4⋯Cl3^iv^	0.94	2.61	3.390 (4)	141
C5—H5⋯N5^v^	0.94	2.47	3.365 (6)	160
C6—H6⋯Cl2^vi^	0.94	2.70	3.315 (5)	124

Symmetry codes: (iii) 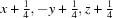; (iv) 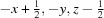; (v) 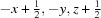; (vi) 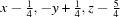.

**Table 3:** Experimental details

Crystal data	
Chemical formula	[Cu_4_Cl_8_(C_4_H_4_N_6_)_3_]
*M*_r_	946.16
Crystal system, space group	Orthorhombic, *F* *d* *d*2
Temperature (K)	213
*a*, *b*, *c* (Å)	28.869 (2), 31.584 (2), 6.2953 (4)
*V* (Å^3^)	5740.1 (7)
*Z*	8
Radiation type	Mo *K*α
μ (mm^−1^)	3.71
Crystal size (mm)	0.18 × 0.15 × 0.14

Data collection	
Diffractometer	Stoe Image plate diffraction system
Absorption correction	Numerical [*X-RED* (Stoe & Cie, 2001 ▸) and *X-SHAPE* (Stoe & Cie, 1999 ▸)]
*T*_min_, *T*_max_	0.548, 0.608
No. of measured, independent and observed [*I* > 2σ(*I*)] reflections	10421, 3323, 3116
*R*_int_	0.027
(sin θ/λ)_max_ (Å^−1^)	0.661

Refinement	
*R*[*F* ^2^ > 2σ(*F* ^2^)], *wR*(*F* ^2^), *S*	0.023, 0.055, 1.02
No. of reflections	3323
No. of parameters	190
No. of restraints	1
H-atom treatment	H-atom parameters constrained
Δρ_max_, Δρ_min_ (e Å^−3^)	0.88, −0.50
Absolute structure	Flack *x* determined using 1309 quotients [(*I* ^+^)−(*I* ^−^)]/[(*I* ^+^)+(*I* ^−^)] (Parsons *et al.*, 2013 ▸)
Absolute structure parameter	−0.010 (9)

Computer programs: *IPDS Software* (Stoe & Cie, 2000 ▸), *SHELXS97* (Sheldrick, 2008 ▸), *SHELXL2018/1* (Sheldrick, 2015 ▸), *DIAMOND* (Brandenburg, 1999 ▸), *WinGX* (Farrugia, 2012 ▸) and *PLATON* (Spek, 2009 ▸).

## supplementary crystallographic information

### Crystal data

**Table:**

[Cu_4_Cl_8_(C_4_H_4_N_6_)_3_]	*D*_x_ = 2.190 Mg m^−^^3^
*M**_r_* = 946.16	Mo *K*α radiation, λ = 0.71073 Å
Orthorhombic, *F**d**d*2	Cell parameters from 8000 reflections
*a* = 28.869 (2) Å	θ = 1.9–28.0°
*b* = 31.584 (2) Å	µ = 3.71 mm^−^^1^
*c* = 6.2953 (4) Å	*T* = 213 K
*V* = 5740.1 (7) Å^3^	Prism, green
*Z* = 8	0.18 × 0.15 × 0.14 mm
*F*(000) = 3696	

### Data collection

**Table:**

Stoe Image plate diffraction system diffractometer	3116 reflections with *I* > 2σ(*I*)
Radiation source: fine-focus sealed tube	*R*_int_ = 0.027
φ oscillation scans	θ_max_ = 28.0°, θ_min_ = 1.9°
Absorption correction: numerical [X-RED (Stoe & Cie, 2001) and X-SHAPE (Stoe & Cie, 1999)]	*h* = −38→35
*T*_min_ = 0.548, *T*_max_ = 0.608	*k* = −41→41
10421 measured reflections	*l* = −7→7
3323 independent reflections	

### Refinement

**Table:**

Refinement on *F*^2^	Secondary atom site location: difference Fourier map
Least-squares matrix: full	Hydrogen site location: inferred from neighbouring sites
*R*[*F*^2^ > 2σ(*F*^2^)] = 0.023	H-atom parameters constrained
*wR*(*F*^2^) = 0.055	*w* = 1/[σ^2^(*F*_o_^2^) + (0.0381*P*)^2^] where *P* = (*F*_o_^2^ + 2*F*_c_^2^)/3
*S* = 1.02	(Δ/σ)_max_ = 0.001
3323 reflections	Δρ_max_ = 0.88 e Å^−^^3^
190 parameters	Δρ_min_ = −0.50 e Å^−^^3^
1 restraint	Absolute structure: Flack *x* determined using 1309 quotients [(*I*^+^)-(*I*^-^)]/[(*I*^+^)+(*I*^-^)] (Parsons *et al.*, 2013)
Primary atom site location: structure-invariant direct methods	Absolute structure parameter: −0.010 (9)

### Special details

**Table:**

Geometry. All esds (except the esd in the dihedral angle between two l.s. planes) are estimated using the full covariance matrix. The cell esds are taken into account individually in the estimation of esds in distances, angles and torsion angles; correlations between esds in cell parameters are only used when they are defined by crystal symmetry. An approximate (isotropic) treatment of cell esds is used for estimating esds involving l.s. planes.

### Fractional atomic coordinates and isotropic or equivalent isotropic displacement parameters (Å^2^)

**Table:**

	*x*	*y*	*z*	*U*_iso_*/*U*_eq_	
Cu1	0.19850 (2)	0.11917 (2)	0.94479 (8)	0.01706 (11)	
Cu2	0.15568 (2)	0.05097 (2)	0.52000 (9)	0.01686 (11)	
Cl1	0.14339 (3)	0.11976 (3)	0.63547 (17)	0.0209 (2)	
Cl2	0.26125 (4)	0.14870 (5)	1.1066 (3)	0.0475 (4)	
Cl3	0.17082 (4)	0.05490 (3)	1.10950 (19)	0.0239 (2)	
Cl4	0.16908 (3)	−0.01996 (3)	0.54022 (18)	0.0218 (2)	
N1	0.23912 (10)	0.08783 (9)	0.7448 (6)	0.0167 (7)	
N2	0.22374 (10)	0.06244 (9)	0.5786 (6)	0.0179 (7)	
N3	0.29808 (10)	0.06871 (9)	0.5595 (6)	0.0170 (7)	
N4	0.40573 (10)	0.09304 (9)	0.3439 (6)	0.0177 (7)	
N5	0.41628 (11)	0.05456 (10)	0.4425 (7)	0.0226 (8)	
N6	0.34333 (10)	0.06931 (9)	0.4869 (6)	0.0155 (7)	
N7	0.08703 (10)	0.04075 (10)	0.4717 (6)	0.0204 (7)	
N8	0.06179 (12)	0.06766 (11)	0.3404 (7)	0.0279 (8)	
N9	0.01831 (10)	0.01434 (9)	0.4350 (6)	0.0200 (7)	
C1	0.28398 (12)	0.09087 (11)	0.7326 (7)	0.0158 (8)	
H1	0.303232	0.105780	0.826934	0.019*	
C2	0.25984 (13)	0.05162 (11)	0.4654 (8)	0.0207 (8)	
H2	0.259496	0.035051	0.341342	0.025*	
C3	0.36206 (12)	0.10152 (11)	0.3721 (7)	0.0171 (7)	
H3	0.346291	0.125570	0.322048	0.021*	
C4	0.37822 (13)	0.04129 (11)	0.5283 (8)	0.0219 (8)	
H4	0.375067	0.016178	0.607163	0.026*	
C5	0.06065 (13)	0.00912 (12)	0.5272 (8)	0.0226 (8)	
H5	0.069354	−0.013471	0.615824	0.027*	
C6	0.02098 (15)	0.05083 (13)	0.3162 (9)	0.0289 (10)	
H6	−0.002904	0.061861	0.231151	0.035*	

### Atomic displacement parameters (Å^2^)

**Table:**

	*U*^11^	*U*^22^	*U*^33^	*U*^12^	*U*^13^	*U*^23^
Cu1	0.0133 (2)	0.01689 (19)	0.0210 (3)	0.00071 (16)	0.00427 (17)	−0.00465 (18)
Cu2	0.01083 (18)	0.01540 (19)	0.0244 (3)	−0.00198 (15)	−0.00067 (18)	−0.00141 (17)
Cl1	0.0206 (4)	0.0200 (4)	0.0222 (6)	0.0052 (3)	−0.0041 (3)	−0.0035 (3)
Cl2	0.0170 (5)	0.0704 (8)	0.0550 (9)	0.0052 (5)	−0.0044 (5)	−0.0457 (7)
Cl3	0.0316 (5)	0.0194 (4)	0.0206 (6)	−0.0048 (3)	0.0037 (4)	0.0015 (3)
Cl4	0.0232 (4)	0.0163 (4)	0.0258 (6)	−0.0001 (3)	−0.0024 (4)	0.0027 (4)
N1	0.0151 (14)	0.0192 (13)	0.016 (2)	−0.0012 (11)	0.0024 (12)	−0.0045 (12)
N2	0.0117 (13)	0.0181 (14)	0.024 (2)	−0.0031 (11)	0.0009 (12)	−0.0045 (12)
N3	0.0120 (14)	0.0175 (14)	0.021 (2)	−0.0010 (10)	0.0038 (12)	−0.0031 (12)
N4	0.0160 (15)	0.0163 (13)	0.021 (2)	−0.0008 (11)	0.0028 (13)	0.0034 (12)
N5	0.0180 (15)	0.0172 (14)	0.033 (2)	0.0018 (11)	0.0052 (14)	0.0047 (14)
N6	0.0107 (12)	0.0178 (13)	0.018 (2)	−0.0019 (10)	0.0038 (11)	−0.0024 (12)
N7	0.0153 (14)	0.0188 (14)	0.027 (2)	−0.0019 (11)	−0.0017 (13)	0.0014 (13)
N8	0.0225 (17)	0.0229 (16)	0.038 (3)	−0.0033 (13)	−0.0041 (15)	0.0103 (15)
N9	0.0127 (14)	0.0174 (14)	0.030 (2)	−0.0037 (11)	−0.0007 (13)	0.0017 (13)
C1	0.0134 (15)	0.0155 (14)	0.018 (2)	−0.0008 (12)	0.0018 (14)	−0.0032 (13)
C2	0.0158 (16)	0.0222 (17)	0.024 (3)	−0.0045 (13)	0.0026 (15)	−0.0087 (16)
C3	0.0139 (16)	0.0178 (15)	0.020 (2)	−0.0007 (12)	0.0026 (14)	−0.0009 (14)
C4	0.0186 (17)	0.0174 (16)	0.030 (2)	0.0007 (13)	0.0035 (16)	0.0010 (16)
C5	0.0184 (17)	0.0202 (16)	0.029 (3)	−0.0025 (13)	−0.0035 (17)	0.0044 (16)
C6	0.022 (2)	0.0262 (19)	0.039 (3)	−0.0040 (15)	−0.0051 (18)	0.0107 (18)

### Geometric parameters (Å, º)

**Table:**

Cu1—N4^i^	1.957 (3)	N4—N5	1.398 (4)
Cu1—N1	1.985 (3)	N5—C4	1.294 (5)
Cu1—Cl2	2.2780 (12)	N6—C3	1.360 (5)
Cu1—Cl3	2.4155 (10)	N6—C4	1.366 (5)
Cu1—Cl1	2.5146 (11)	N7—C5	1.304 (5)
Cu2—N7	2.031 (3)	N7—N8	1.392 (5)
Cu2—N2	2.032 (3)	N8—C6	1.302 (5)
Cu2—Cl4	2.2769 (9)	N9—C5	1.363 (5)
Cu2—Cl1	2.3185 (10)	N9—C6	1.376 (5)
Cu2—Cl3^ii^	2.6238 (13)	N9—N9^iii^	1.392 (6)
N1—C1	1.301 (5)	C1—H1	0.9400
N1—N2	1.391 (5)	C2—H2	0.9400
N2—C2	1.308 (5)	C3—H3	0.9400
N3—C1	1.357 (5)	C4—H4	0.9400
N3—C2	1.364 (5)	C5—H5	0.9400
N3—N6	1.384 (4)	C6—H6	0.9400
N4—C3	1.301 (5)		
			
N4^i^—Cu1—N1	168.82 (15)	C3—N4—Cu1^v^	123.5 (3)
N4^i^—Cu1—Cl2	92.14 (10)	N5—N4—Cu1^v^	127.3 (2)
N1—Cu1—Cl2	91.04 (9)	C4—N5—N4	106.4 (3)
N4^i^—Cu1—Cl3	95.62 (10)	C3—N6—C4	107.0 (3)
N1—Cu1—Cl3	92.79 (10)	C3—N6—N3	124.1 (3)
Cl2—Cu1—Cl3	114.53 (6)	C4—N6—N3	128.6 (3)
N4^i^—Cu1—Cl1	88.14 (11)	C5—N7—N8	108.7 (3)
N1—Cu1—Cl1	83.47 (10)	C5—N7—Cu2	130.7 (3)
Cl2—Cu1—Cl1	147.82 (6)	N8—N7—Cu2	120.2 (2)
Cl3—Cu1—Cl1	97.43 (4)	C6—N8—N7	107.1 (3)
N7—Cu2—N2	177.81 (15)	C5—N9—C6	106.4 (3)
N7—Cu2—Cl4	91.02 (9)	C5—N9—N9^iii^	127.0 (3)
N2—Cu2—Cl4	90.06 (9)	C6—N9—N9^iii^	126.0 (3)
N7—Cu2—Cl1	92.67 (10)	N1—C1—N3	107.9 (3)
N2—Cu2—Cl1	85.63 (9)	N1—C1—H1	126.0
Cl4—Cu2—Cl1	158.52 (5)	N3—C1—H1	126.0
N7—Cu2—Cl3^ii^	91.29 (12)	N2—C2—N3	107.7 (4)
N2—Cu2—Cl3^ii^	90.54 (11)	N2—C2—H2	126.1
Cl4—Cu2—Cl3^ii^	94.20 (4)	N3—C2—H2	126.1
Cl1—Cu2—Cl3^ii^	106.86 (4)	N4—C3—N6	107.7 (3)
Cu2—Cl1—Cu1	97.99 (4)	N4—C3—H3	126.2
Cu1—Cl3—Cu2^iv^	121.17 (4)	N6—C3—H3	126.2
C1—N1—N2	108.4 (3)	N5—C4—N6	109.7 (3)
C1—N1—Cu1	126.1 (3)	N5—C4—H4	125.2
N2—N1—Cu1	125.2 (2)	N6—C4—H4	125.2
C2—N2—N1	107.8 (3)	N7—C5—N9	108.5 (4)
C2—N2—Cu2	128.7 (3)	N7—C5—H5	125.8
N1—N2—Cu2	123.2 (2)	N9—C5—H5	125.8
C1—N3—C2	108.1 (3)	N8—C6—N9	109.2 (4)
C1—N3—N6	122.8 (3)	N8—C6—H6	125.4
C2—N3—N6	128.7 (3)	N9—C6—H6	125.4
C3—N4—N5	109.2 (3)		
			
C1—N1—N2—C2	−1.8 (4)	Cu2—N2—C2—N3	175.9 (3)
Cu1—N1—N2—C2	171.8 (3)	C1—N3—C2—N2	−0.9 (5)
C1—N1—N2—Cu2	−176.4 (3)	N6—N3—C2—N2	−173.2 (3)
Cu1—N1—N2—Cu2	−2.8 (4)	N5—N4—C3—N6	−0.2 (5)
C3—N4—N5—C4	−0.4 (5)	Cu1^v^—N4—C3—N6	−178.0 (3)
Cu1^v^—N4—N5—C4	177.3 (3)	C4—N6—C3—N4	0.7 (5)
C1—N3—N6—C3	−78.1 (5)	N3—N6—C3—N4	175.6 (4)
C2—N3—N6—C3	93.2 (5)	N4—N5—C4—N6	0.8 (5)
C1—N3—N6—C4	95.6 (5)	C3—N6—C4—N5	−1.0 (5)
C2—N3—N6—C4	−93.1 (6)	N3—N6—C4—N5	−175.5 (4)
C5—N7—N8—C6	−1.5 (5)	N8—N7—C5—N9	0.2 (5)
Cu2—N7—N8—C6	172.0 (3)	Cu2—N7—C5—N9	−172.4 (3)
N2—N1—C1—N3	1.2 (4)	C6—N9—C5—N7	1.2 (5)
Cu1—N1—C1—N3	−172.3 (3)	N9^iii^—N9—C5—N7	172.8 (3)
C2—N3—C1—N1	−0.2 (4)	N7—N8—C6—N9	2.2 (6)
N6—N3—C1—N1	172.6 (3)	C5—N9—C6—N8	−2.1 (6)
N1—N2—C2—N3	1.6 (4)	N9^iii^—N9—C6—N8	−173.9 (4)

Symmetry codes: (i) *x*−1/4, −*y*+1/4, *z*+3/4; (ii) *x*, *y*, *z*−1; (iii) −*x*, −*y*, *z*; (iv) *x*, *y*, *z*+1; (v) *x*+1/4, −*y*+1/4, *z*−3/4.

### Hydrogen-bond geometry (Å, º)

**Table:**

*D*—H···*A*	*D*—H	H···*A*	*D*···*A*	*D*—H···*A*
C1—H1···Cl1^vi^	0.94	2.74	3.528 (4)	142
C1—H1···Cl2	0.94	2.53	3.052 (4)	115
C2—H2···Cl4^vii^	0.94	2.84	3.518 (4)	130
C3—H3···Cl2^ii^	0.94	2.90	3.672 (4)	140
C3—H3···N8^vi^	0.94	2.66	3.242 (5)	121
C4—H4···Cl3^vii^	0.94	2.61	3.390 (4)	141
C5—H5···N5^viii^	0.94	2.47	3.365 (6)	160
C6—H6···Cl2^ix^	0.94	2.70	3.315 (5)	124

Symmetry codes: (ii) *x*, *y*, *z*−1; (vi) *x*+1/4, −*y*+1/4, *z*+1/4; (vii) −*x*+1/2, −*y*, *z*−1/2; (viii) −*x*+1/2, −*y*, *z*+1/2; (ix) *x*−1/4, −*y*+1/4, *z*−5/4.
